# The complete chloroplast genome of *Vaccinium duclouxii*, an endemic species in China

**DOI:** 10.1080/23802359.2019.1624644

**Published:** 2019-07-11

**Authors:** Xin Chen, Qingzhong Liu, Wei Guo, Hairong Wei, Jiawei Wang, Dongzi Zhu, Yue Tan

**Affiliations:** a Shandong Provincial Key Laboratory of Fruit Tree Biotechnology Breeding, Shandong Institute of Pomology, Shandong Academy of Agricultural Sciences, Taian, Shandong, P. R. China;; b Taishan Academy of Forestry Sciences, Taian, Shandong, P. R. China

**Keywords:** *Vaccinium duclouxii*, chloroplast genome, Illumina sequencing

## Abstract

*Vaccinium duclouxii* is an endemic species in China, which is distributed in Sichuan and Yunnan province of China. The chloroplast (cp) genome of *V. duclouxii* is 168,953 bp in size containing 123 unique genes, including 8 rRNA genes, 38 tRNA genes, and 77 protein-coding genes (PCGs). Phylogenetic analysis exhibited that *V. duclouxii* and *V. macrocarpon* were most related to *Arbutus unedo*.

*Vaccinium duclouxii*, an endemic species in China, is a plant belonging to the family Vacciniaceae. The evergreen shrub or small tree reaches 1-5 m in height and rarely goes over 10 m. It is very similar to *V. mandarinorum* and has flowers with very short pedicels. *V. duclouxii* is distributed in Sichuan and Yunnan province of China (Fang 1991). It grows in evergreen broad-leaved forest, pine forest, and the hillside shrubbery. The altitude of its natural mountain habitat is between 1550 and 2600 m. The berries turn purple-black when they are fully ripe at ripening stages from July to November. The genomic sequence information is urgently needed to promote molecular evolution, systematics research, conservation and utilization of *V. duclouxii*. The objectives of the present study were to reconstruct the cp genome of *V. duclouxii* and assess phylogenetic relationships.

Leaves were sampled from a mature *V. duclouxii* tree at Jiuhe, Yulong Naxi Autonomous County, Lijiang, Yunnan, China (26°37′9.08″N, 99°56′41.08″E), and chilled with liquid nitrogen immediately. The voucher specimen (accession no. TS_2019_Yulong_Taian) was stored at –80 °C in Shandong Institute of Pomology (SDIP). Genomic DNA (gDNA) was obtained from homogenized leaf tissues using a modified CTAB protocol (Doyle and Doyle 1987). The quantity and quality of the purified gDNA were detected by Nanodrop 8000 and via the Agilent 2100 Bioanalyzer. A library with 350 bp fragments inserted was constructed with 1 μg purified DNA and high-throughput sequenced with paired end (PE) reads of 2 × 150 bp on Illumina Hiseq 2500 platform. Raw reads were filtered and trimmed to remove low quality and contaminated reads by trim_galore v0.4.4. Total 8.8 Gb of clean data were aligned to the *V. macrocarpon* complete cp genome (GenBank no. JQ757046) as a reference using bowtie2 v2.2.4 (Langmead and Salzberg 2012) and assembled with SPAdes v3.10.1 (Bankevich et al. 2012). The final cp genome was annotated using DOGMA (Boore et al. 2004), HMMER 3.1b2 (Finn et al. 2011) and ARAGORN v1.2.38 (Laslett and Canback 2004).

The cp genome of *V. duclouxii* (GenBank no. MK816300) is 168,953 bp in size with total AT content 63.3%. It contains a 3042 bp small and 106,285 bp large single-copy regions with AT contents 71.3 and 64.1%, respectively, and two 29,813 bp inverted repeat regions with AT content 61.3%. In the cp genome of *V. duclouxii,* there are 123 unique genes, including 8 rRNA genes, 38 tRNA genes and 77 PCGs. Eight genes harbour one intron each, while a PCG *ycf3* harbour two introns.

To perform the molecular phylogenetic analysis, 15 published complete cp genomes were aligned by MAFFT v7.307 (Katoh and Standley 2013). Finally, a maximum likelihood (ML) tree was constructed using RAxML v.7.2.6 with the GTRGAMMA model (Stamatakis 2006). The ML phylogenetic tree indicated that *V. duclouxii* and *V. macrocarpon* were most related to *Arbutus unedo* (Figure 1), which was consistent with the most recent report (Bao 2018).

**Figure 1.**
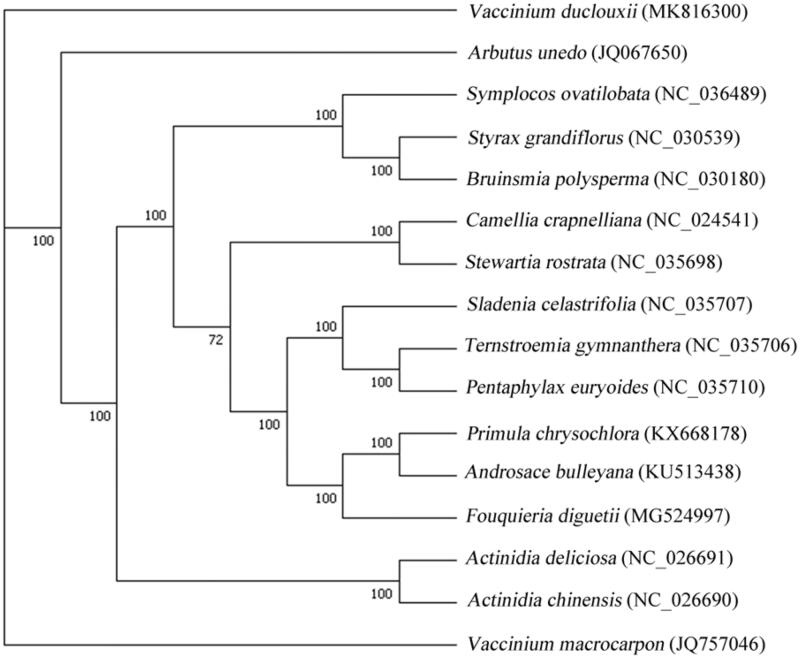
Phylogenetic tree based on 16 complete cp genome sequences. The bootstrap support values are shown next to the branches.
